# Clinical presentation of pertussis in fully immunized children in Lithuania

**DOI:** 10.1186/1471-2334-5-40

**Published:** 2005-05-27

**Authors:** Irena Narkeviciute, Ema Kavaliunaite, Genovaite Bernatoniene, Rimantas Eidukevicius

**Affiliations:** 1 Center of Pediatrics, Clinic of Children's Diseases, Vilnius University, Santariskiu 4, 08406 Vilnius, Lithuania; 2 Laboratory of Microbiology, Vilnius University Children's Hospital, Santariskiu 4, 08406 Vilnius, Lithuania; 3 Department of Mathematics and Informatics, Vilnius University, Naugarduko 24, 03225 Vilnius, Lithuania

## Abstract

**Background:**

In Lithuania, the vaccination coverage against pertussis is high. Nevertheless, there is a significant increase in pertussis cases in fully immunized children. The aim of our study was to determine the frequency of classical symptoms of laboratory confirmed pertussis and describe its epidemiology in children fully vaccinated against pertussis.

**Methods:**

From May to December 2001, 70 children aged 1 month to 15 years, suffering from prolonged cough were investigated in the Centre of Paediatrics, Vilnius University Children's Hospital. The collected information included personal data, vaccination history, clinical symptoms of the current illness, and treatment before hospitalization. At the admission to the hospital blood samples were taken from all studied children for *Bordetella pertussis *IgM and IgA.

**Results:**

A total of 53 (75.7%) of the 70 recruited patients with prolonged cough showed laboratory evidence of pertussis. 32 of them were fully vaccinated with whole cell pertussis vaccine (DTP). The age of fully vaccinated patients varied from 4 to 15 years (average 10.9 ± 3.1; median 11). The time period between the last vaccination dose (fourth) and the clinical manifestation of pertussis was 2.6–13 years (average 8.9 ± 3.0; median 9). More than half of the children before the beginning of pertussis were in contact with persons suffering from long lasting cough illness in the family, school or day-care center. The mean duration from onset of pertussis symptoms until hospitalization was 61.4 ± 68.3 days (range, 7 to 270 days; median 30). For 11 patients who had had two episodes (waves) of coughing, the median duration of cough was 90 days, and for 21 with one episode 30 days (p < 0.0002). Most of the children (84.4%) had paroxysmal cough, 31.3% had post-tussive vomiting, 28.1% typical whoop, and 3.1% apnea. Only 15.6% children had atypical symptoms of pertussis.

**Conclusion:**

Fully vaccinated children fell ill with pertussis at the median of 11 years old, 9 years following pertussis vaccination. More than half of the children could catch pertussis at home, at school or day-care center. Clinical picture of pertussis in previously immunized children is usually characterized by such classical symptoms as prolonged and paroxysmal cough, rarely by whopping and post-tussive vomiting, and very rarely by apnea.

## Background

Pertussis is a highly communicable, vaccine-preventable respiratory disease. The incidence of pertussis has been greatly reduced by massive vaccination. Nevertheless, there is a significant increase in pertussis cases in older children, adolescents and adult people [1-4]. Improved diagnosis, awareness of pertussis, genetic *Bordetella pertussis *changes and waning of vaccine-induced immunity are the possible reasons for increased incidence of pertussis [1-5]. In the USA the incidence of vaccine-preventable diseases such as measles, rubella, mumps, diphtheria, tetanus has been greatly reduced in the last 15 years. However, the incidence of pertussis cases increased more than twice: 8296 reported cases in 2002 versus 3450 in 1988 [6]. The age distribution of patients with pertussis in the USA in 1994–1996 and 1997–2000 has changed. During the last period, the incidence of pertussis among infants increased by 11%, in children aged 1–4 years decreased to 8%, remained stable for children aged 5–9 years and among adolescents and adults increased by about 60% [7].

In Lithuania immunization of infants and children against pertussis has been introduced since 1956 and massive vaccination started in 1961. According to our standard vaccination schedule, pertussis whole-cell vaccine incorporated in diphtheria-tetanus-pertussis (DTP) vaccine is offered at 3, 4.5 and 6 months of age with a booster dose only at 18 months of age. In 1991, the vaccine coverage among children aged 1 year was 73.2%, whereas this percentage has been increasing and since 1996 reached above 90% (93.6% in 2000, 94.6% in 2001). 35% of all pertussis cases were diagnosed in vaccinated children (at least three DTP vaccine doses) during the period from 1991 to 1995, 33.4% of the cases from 1996 to 2000 and 43.2% in 2001.

Clinical presentation of pertussis in unvaccinated children had been extensively described by several authors [8,9]. The disease in these patients is usually typical and often severe. Data of the clinical course of pertussis in fully immunized children is usually atypical and generally mild [10].

The aim of our study was to determine the frequency of classical symptoms of laboratory confirmed pertussis and describe its epidemiology in fully vaccinated children.

## Methods

From May to December 2001, 70 children aged 1 month to 15 years with prolonged cough (duration ≥ 14 days) and siblings with shorter duration cough (but not less than 7 days) were hospitalized and investigated at Vilnius University Children's Hospital, Centre of Paediatrics. The patients were referred to the hospital by general practitioners or pediatricians, because detailed investigation of the children with prolonged cough of unknown etiology was only available in the hospital. The data regarding to the patient's age, vaccination history, clinical symptoms and signs of the current illness, previous treatment was collected on to computer database. Single blood samples were taken from all the children upon admission and sent for detection of specific immunoglobulin (Ig) IgM and IgA antibodies to *B. pertussis *by an enzyme-linked immunosorbent assay (ELISA). Serological tests were performed and estimated according to the manufacturer's instructions (Labsystems, Finland). Specimens for *B. pertussis *and *B. parapertussis *culture were not obtained because of the late illness stage and received previous antimicrobial therapy before hospitalization. Classical symptoms of pertussis were defined as a prolonged cough lasting two weeks or more, paroxysmal cough, inspiratory whoop, post-tussive vomiting and apnea. Confirmed case of pertussis was defined as an episode of cough lasting 7 days or more and positive anti-*B. pertussis *IgM or IgA or both levels. Two groups of our studied patients were compared. One group of children with prolonged cough who had two episodes (waves) of successive coughing (when first episode was not ended, it means that cough become more intensified and coughing paroxysm renewed) was compared to the second group who had only one episode of prolonged cough.

The descriptive values were expressed as means, medians and standard deviations (SD) or percentages. Two-sided Wilcoxon test for independent samples was used to compare the groups. A *p *value less than 0.05 were considered statistically significant.

## Results

Of the 70 studied children, 53 (75.7%) showed a serological evidence of pertussis. Out of 53 patients, 21 (39.6%) were partially vaccinated or unvaccinated at all and 32 (60.4%) received complete vaccination. Out of 32 fully vaccinated patients, positive *B. pertussis *IgM antibodies were found in 6, IgA in 3 and both IgM and IgA in 23 patients.

We present epidemiological and clinical analysis of 32 children with confirmed pertussis who received 4 doses whole-cell of pertussis vaccine. Only eight (25%) of 32 fully vaccinated children were referred to the hospital with suspicion of pertussis, other referral diagnosis were bronchitis for 15 children, tracheobronchitis – 4, bronchopneumonia – 4, asthma exacerbation – 1. The characteristics of the children with pertussis are listed in Table 1. There was a difference in gender distribution: male 34.4% and female 65.6%. The age of the patients was from 4 to 15 years (average 10.9 ± 3.1; median 11). Most of the children (75.0%) were older than 9 years. The time period between the last (fourth) vaccination dose and the clinical manifestation of pertussis was 2.6–13 years (average 8.9 ± 3.0; median 9). None of the children had a history of pertussis. Seventeen (53.1%) children before they got ill had been in contact with person suffering from long-lasting cough, 12 of them at home (all of them with siblings and three with one of the parents) and 5 at school or day-care centre. In our study, 9 cases of pertussis were observed in four families: in three families 2 cases in each and in one family 3 cases. Before hospitalization 28 (87.5%) patients had received antibiotic therapy effective against *B. pertussis *(amoxicillin, ampicillin, macrolide).

**Table 1:** Characteristics of fully immunized children with pertussis

Characteristics	n = 32	%
Age (years):		
4–5	2	6.2
6–8	6	18.8
9–11	9	28.1
12–15	15	46.9

Gender:		
Male	11	34.4
Female	21	65.6

Antibiotic treatment		
before investigation	28	87.5

The rate of pertussis symptoms among fully immunized children is shown in Table 2. The mean duration from the onset of pertussis symptoms until hospitalization was 61.4 ± 68.3 days (range, 7 to 270 days; median 30). Most of the children (22; 68.8%) had cough that lasted more than three weeks. For 11 (34.4%) children who had had two episodes (waves) of successive coughing, the mean duration of cough was 127.3 ± 81.7 days (range, 40 to 270 days; median 90), and for 21 (65.6%) with one episode 26.9 ± 16.2 days (range, 7 to 70 days; median 30). The difference was statistically significant (Wilcoxon test, p < 0.0002).

**Table 2:** Clinical symptoms of pertussis in fully immunized children

Symptoms	n	%
Cough	32	100

Duration of coughing (days):		
7–14	7	21.9
15–21	3	9.3
22≥	22	68.8

Paroxysmal cough	27	84.4

Whooping	9	28.1

Post-tussive vomiting	10	31.3

Apnea	1	3.1

The prevalence of other classical symptoms of pertussis was following: paroxysmal cough 27 (84.4%), post-tussive vomiting 10 (31.3%), 9 (28.1%) showed a typical "whoop" and 1 (3.1%) apnea. Five (15.6%) children presented without classical symptoms of pertussis. The duration of their cough was 14 days and more, however, they did not have any coughing paroxysms, whooping, post-tussive vomiting or apnea.

## Discussion

Lithuanian Centre of Infectious Disease Control and Prophylaxis reported 162 cases of pertussis in 2001, 43.2% of them were vaccinated against pertussis. Almost half of all patients (47.5%) with pertussis were aged 7–15 years. Our study analyzed the epidemiological data and clinical presentation of 32 fully immunized children with laboratory-confirmed pertussis hospitalized in the Centre of Paediatrics, Vilnius University Children's Hospital from May to December 2001. Detailed analysis of our study data showed that fully vaccinated children got ill with pertussis at the median of 11 years old (range, 4 to 15), after 9 years (range, 2.6 to 13) following four doses vaccination with DTP vaccine. According to the data reported in 1998 in Finland where pertussis vaccination has been in practice for more than 40 years and a four-dose schedule is completed before a child is 2 years old, over the last decade the reported cases of pertussis increased twice and most of the patients were schoolchildren [4]. It is known that vaccine-induced immunity wanes and after 5–10 years makes the vaccinated host vulnerable to infection [11,12]. Reported study from Poland [13] revealed protective immunity against pertussis in 70% of six years old children, in 68% of seven and only in 45% of eight years old children. Thus, waning of immunity, leads to a growing population of pertussis-susceptible older children, adolescents and an increasing proportion of disease cases in these age groups. According to the literature data vaccination with whole-cell vaccines induces protective immunity lasting 6–8 years [11,14], whereas protective immunity induced by vaccination with acellular pertussis vaccine lasts 4–6 years and longer [15-17]. Torvaldsen and McIntyre [18] showed that the fifth dose of pertussis vaccine given at 4–5 years of age reduced the incidence of pertussis in older children.

Many studies have reported the importance of household and school contacts for pertussis infection. The results of the study performed in France during 1993 and 1994 showed that the source of pertussis was a close contact with adult in 46% of identifiable cases, with sibling in 42% of cases and with other family members or friends in 11% of the cases [19]. More recently, in a household contact study in the USA, 53% of people who were identified as the primary source of pertussis infection were aged 13 years or older and 26% were aged 30 years or older [20]. Our study data showed that more than half of the patients had been in contact with persons who had prolonged cough: mostly in families and rarely at schools or day-care centers.

Clinical presentation of pertussis in fully immunized children become talking-point in the clinical practice. Various factors such as patient's age, gender, antibiotic treatment and especially vaccination status may influence clinical presentation of pertussis. Vaccination significantly changes the clinical presentation of pertussis [8-10,21,22]. Prolonged cough is one of the classical symptoms of pertussis. Before investigation the median duration of cough in all our patients was one month. Tozzi et al. [8] in a study of 788 laboratory confirmed cases of pertussis had demonstrated that the duration of cough in vaccinated children was about one month, while in unvaccinated was two times longer. Two episodes of cough and significantly prolonged cough (median 90 days) documented in our study may be accounted for co-infection of *B. pertussis *with atypical pathogens. Hallander et al. from Sweden [23] demonstrated that the duration of cough increased when more than one agent was detected. A median cough period was 51 days for *B. pertussis *and 60 days for co-infections with *M. pneumoniae *or *C. pneumoniae*.

Unvaccinated children more frequently presented the full spectrum of classical pertussis symptoms than vaccinated children. German study [9] has shown that 90.2% of unvaccinated patients (mean age 4.3 years) had paroxysmal cough, 78.9% whooping and 53.3% post-tussive vomiting. The frequency of paroxysmal cough in fully vaccinated children (median age 11 years) in our study was similar (84.4%), but post-tussive vomiting and whooping was more rare (accordingly 31.3% and 28.2%). Only one 11 years old patient had apnea. The results of our study coincide to those reported from Canada [21]. Of the 103 immunized children (< 5 years of age), 68% developed paroxysmal cough within the first week of their illness and 88% had persistent paroxysmal cough for more than three weeks. Cough generally lasted 16–91 days (median 48). A recent study conducted in Israel [10] has documented the clinical manifestation of pertussis in previously immunized children and young adults (median age 9 years). 21% of the patients had paroxysmal cough, 13% showed post-tussive vomiting, 7% apnea and 6% had classic whoop. The classical symptoms of our studied patients therefore were more frequent compared with Israeli patients. However, we should also take into account other fully vaccinated children who had mild pertussis, but were not referred to the hospital and were not recruited in our study.

Some authors have reported that antibiotic treatment has reduced the severity of pertussis and the duration of cough, especially when they have been started at the beginning of the disease [21]. In opposite, the other studies have demonstrated that antibiotic treatment does not influence on the course of pertussis and is not effective [24,25]. Tozzi et al. [8] has showed that children treated with antibiotics had cough which lasted 6 to 11 days longer and spasmodic cough 4 to 13 days longer than untreated patients. Authors concluded that antibiotic therapy may be a marker of severe disease. We had no possibility to evaluate the role of antibiotics on to the duration of the cough in our patients, because most of our children had received antibiotics before examination.

The results of our study showed that prolonged cough in fully vaccinated children are frequently associated with paroxysmal cough and rarely with other symptoms such as whooping, post-tussive vomiting, apnea. Only 15.6% of the patients with a long duration of cough had no classical symptoms of pertussis. Thus, it is very difficult to suspect pertussis in this group of children in the absence of epidemiological data. Our data suggest that pertussis should be considered in the differential diagnosis of prolonged cough in fully vaccinated children.

## Conclusion

Pertussis is a common disease in Lithuania as well as in many other countries with high vaccination coverage. About half of children with pertussis in Lithuania in 2001 were aged 7 to14 years. Fully vaccinated children fell ill with pertussis at the median of 11 years old, 9 years following DTP vaccination. More than half of the children could catch pertussis at home, at school or at day-care center. Clinical picture of pertussis in previously immunized children is usually characterized by such classical symptoms as prolonged and paroxysmal cough, rarely by whopping and post-tussive vomiting, and very rarely by apnea. This study provides useful information for the clinicians about the epidemiology and clinical data of pertussis in fully vaccinated children.

## Competing interests

The author(s) declare that they have no competing interests.

## Authors' contributions

IN conceived of the study, supervised the design and execution of the study, performed the preliminary and final data analysis, drafted and wrote the manuscript.

EK participated in the design of the study, data assessment, preliminary and final data analysis and writing the manuscript.

GB performed the serologic tests, participated in writing the manuscript.

RE performed the statistical analysis, participated in writing the manuscript.

## Pre-publication history

The pre-publication history for this paper can be accessed here:


